# The prevalence of depressive and anxiety symptoms among first-year and fifth-year medical students during the COVID-19 pandemic: a cross-sectional study

**DOI:** 10.1186/s12909-023-04387-x

**Published:** 2023-06-06

**Authors:** Abdullah Alshehri, Badr Alshehri, Omar Alghadir, Abdullah Basamh, Meshari Alzeer, Mohammed Alshehri, Sameh Nasr

**Affiliations:** 1 Department of Surgery, College of Medicine, King Saud University, Riyadh, Saudi Arabia; 2 College of Medicine, King Saud University, Riyadh, Saudi Arabia

**Keywords:** Depression, Anxiety, Medical students, COVID-19

## Abstract

**Background:**

Medical students have higher risk of psychological disorders due to the relatively stressful environment. Educators are becoming increasingly aware of the impact of stresses on the students general well-being. The objective of the current study was to examine the prevalence of and risk factors for depressive and anxiety symptoms among first-year and fifth-year medical students. Additionally, we aimed to determine whether the COVID-19 pandemic has affected students’ mental well-being.

**Methods:**

A cross-sectional study was performed at the College of Medicine at King Saud University between September 2020 and January 2021. The target population was first-year and fifth-year medical students. Depressive symptoms were screened using the 9-item Patient Health Questionnaire (PHQ-9), while anxiety symptoms were screened using the 7-item Generalized Anxiety Disorder assessment (GAD-7). Students were also directly asked about the effect of the COVID-19 pandemic on their mental well-being. Outcomes were compared between groups using the chi-squared test and Student’s t test. Multivariate logistic regression analysis was performed to identify factors associated with depressive and anxiety symptoms.

**Results:**

A total of 182 medical students were included. Depressive symptoms (52.9% versus 35.8%, *p* = 0.020) and anxiety symptoms (35.6% versus 26.3%, *p* = 0.176) were higher in the first-year students than in the fifth-year students. Approximately 19.2% of the students were worried about acquiring COVID-19, 49.4% were worried about academic performance, and 30.8% were feeling sad, depressed or anxious during the COVID-19 pandemic. Independent risk factors for depressive symptoms included having concomitant anxiety, being worried about acquiring COVID-19, being worried about academic performance, and feeling sad, depressed or anxious. Independent risk factors for anxiety included having a lower grade point average and having concomitant depressive symptoms.

**Conclusion:**

Medical students have an alarmingly high prevalence of depressive and anxiety symptoms, which might have been negatively impacted by the COVID-19 pandemic. There is a need for a special mental health program targeting new and current medical students.

**Supplementary Information:**

The online version contains supplementary material available at 10.1186/s12909-023-04387-x.

## Background

Anxiety and depressive disorders are the most common mental disorders worldwide [1, 2]. Approximately 284 million people suffer from anxiety disorders, and 264 million people suffer from depressive disorders worldwide [1]. Depressive disorders and, to a lesser extent, anxiety disorders are listed among the top contributors to the overall global burden of disease and disability, especially among women [1]. Both diseases cause considerable negative impacts on quality of life, including the physical and psychosocial domains [3].

The mental health of medical students has received increasing attention in recent years. Several studies from Arabic and Western countries have indicated that medical students suffer from higher levels of anxiety, depression, and psychological distress than their peers in other specialties [4–7]. Medical education can be stressful due to academic pressure, sleep deprivation, a negative educational environment, limited faculty support, financial concerns, limited leisure time, and emotionally stressful experiences due to exposure to sick and dying patients [8, 9]. Some studies have shown that the impact of these challenges on mental health is higher among newly joined students who face a sudden and major change in their lifestyle [10, 11]. Examining first-year and fifth-year students may provide insight regarding the difference between both groups, as there are several possible confounding factors between them, such as the experience level, level of maturity, and ability to seek support.

The emergence of the COVID-19 pandemic represents a compelling new challenge for the mental health and psychological well-being of the whole world population and medical students in particular [12–16]. Estimating the burden of depressive and anxiety symptoms during the pandemic is critical for planning student mental health services [12–14]. Unfortunately, psychological data such as anxiety and depressive symptoms for medical students during the COVID-19 pandemic are lacking in Saudi Arabia [17]. The objective of the current study was to examine the prevalence of and risk factors for depressive and anxiety symptoms among first-year and fifth-year medical students. Additionally, we determined whether the students felt that the COVID-19 pandemic had affected their mental well-being.

## Methods

### Setting

The current study was conducted at the College of Medicine of King Saud University in Riyadh, Saudi Arabia. The college was established more than 40 years ago as the first medical college in the country. The college hosts more than 1400 male and female medical students.

### Study design and ethical approval

A cross-sectional study was carried out between September 2020 and January 2021. The study obtained all required ethical approvals from the institutional review board at the College of Medicine, King Saud University (No. E20-5302). The students were required to provide consent to join the study. The consent emphasized voluntary participation, confidential handling of data, no sensitive data collection, and the anonymous nature of data analysis.

### Population

The target population was first-year and fifth-year medical students during the 2020–2021 academic year. Students with previously diagnosed psychiatric disorders were also included.

### Sample size

It was estimated that 180 students (90 from each year) are required to detect a 20% difference in study outcomes (depressive and anxiety symptoms) between the two years using 80% power and a 95% level of significance. Given the actual number of medical students at King Saud University in the target years, the calculated sample size would be sufficient to detect 40% study outcomes (depression and/or anxiety) with 10% confidence in each group. The sample was collected using convenience sampling. Students were recruited randomly from classrooms, break areas, and building courtyards. We decided to compare first-year and fifth-year students because they represent the beginning and the end of undergraduate education in the College of Medicine. Therefore, their comparison is more likely to show differences in the measured outcomes due to several factors that distinguish between both groups, such as the experience level, level of maturity, ability to handle stress and seek support when needed, etc.

### Data collection tool

A self-administered online questionnaire (using Google Forms) was developed and included sociodemographic characteristics, academic performance, and perceived probable COVID-19 impact on students’ psychology. The questionnaire was reviewed by three experts to assess face and content validity. The experts were clinicians working in the student clinic with experience in assessing the physical and psychological health of medical students. A pilot study was conducted with 10 students to check the applicability and clarity of the questions. The pilot study showed positive feedback to the questions. Additionally, previously validated psychometric tools were used to assess the study outcomes (depressive and anxiety symptoms) [18, 19]. Students were directly asked about their feelings and academic performance during the pandemic. All included students were asked to sign informed consent forms before starting the questionnaire. Data confidentiality was protected through an online self-administered collection method, deidentification, encryption, password protection and limited access to the research team only. The questionnaire is provided as a supplementary document.

### Psychometric tools

Depressive symptoms were screened using the 9-item Patient Health Questionnaire (PHQ-9), while anxiety symptoms were screened using the 7-item Generalized Anxiety Disorder assessment (GAD-7) [18, 19]. Both tools were developed and validated to establish provisional diagnoses for depressive and anxiety symptoms (respectively) and follow response to intervention and treatment in accordance with standard definitions of the Diagnostic and Statistical Manual of Mental Disorders (DSM-IV) [20]. According to the clinical literature, the PHQ-9 score of ≥ 10 indicating clinical depression, and the GAD-7 score of ≥ 10 indicating clinical anxiety [18–20].

### Statistical analysis

Data are presented as frequencies and percentages for categorical data and mean and standard deviation (SD) for continuous data. Sociodemographic characteristics, academic performance, COVID-19-related questions, and study outcomes were compared between the study groups (first year and fifth year). Additionally, the previous factors were examined to detect the univariate risk factors for depressive and anxiety symptoms. Chi-squared or Fisher’s exact tests, as appropriate, were used to examine differences in categorical variables, while Student’s t test was used to examine differences in continuous variables. To identify factors independently associated with depressive and anxiety symptoms, multivariate logistic regression analysis models were run after adjusting for the variables that were potentially associated with awareness in univariate analysis (with *p* < 0.20). Backwards elimination was used to exclude nonsignificant variables from the model. To simplify the analysis of COVID-related questions, responses of (always) and (most of the time) were analysed as (yes) and (not at all) and (sometimes), which were analysed as (no). A *P* value < 0.05 was considered significant. SPSS (Version 25.0. Armonk, NY: IBM Corp) was used for all statistical analyses.

## Results

A total of 182 medical students were included in the current study. Table 1 shows the sociodemographic and academic characteristics by academic year of the students. Compared with fifth-year students, first-year students had significantly younger age (19.1 ± 1.1 versus 23.1 ± 0.6 years, *p* < 0.001), significantly better grade point average (GPA) (83.9% versus 43.2% for GPA 4.50 to 5.00, *p* < 0.001), significantly more frequent successful academic years (97.7% versus 90.5%, *p* = 0.042), significantly more frequent within-family living status (94.3% versus 88.4%, 0.037), and significantly more frequent sedentary (not active) lifestyle (29.9% versus 15.2%, *p* = 0.009). There were no significant differences between students in the two academic years in gender, use of psychological therapy, sleeping duration, single social status, and current smoking.

**Table 1:** Sociodemographic and academic characteristics by the academic year of medical students

	**First-year** **(*N* = 87)**	**Fifth-year** **(*N* = 95)**	**Total** **(*N* = 182)**	***p*-value**
**Age**	19.1 ± 1.1	23.1 ± 0.6	21.2 ± 2.2	< 0.001
**Gender**				
Male	54 (62.1%)	46 (48.4%)	100 (54.9%)	0.065
Female	33 (37.9%)	49 (51.6%)	82 (45.1%)	
**GPA**				
3.00 to 3.99	3 (3.4%)	27 (28.4%)	30 (16.5%)	< 0.001
4.00 to 4.49	11 (12.6%)	27 (28.4%)	38 (20.9%)	
4.50 to 5.00	73 (83.9%)	41 (43.2%)	114 (62.6%)	
**Previously failed academic years**				
No	85 (97.7%)	86 (90.5%)	171 (94.0%)	0.042
Yes	2 (2.3%)	9 (9.5%)	11 (6.0%)	
**Current psychological therapy**				
No	74 (85.1%)	70 (73.7%)	144 (79.1%)	0.059
Yes	13 (14.9%)	25 (26.3%)	38 (20.9%)	
**Currently living status**				
Alone	3 (3.4%)	11 (11.6%)	14 (7.7%)	0.037
With family	82 (94.3%)	84 (88.4%)	166 (91.2%)	
Others	2 (2.3%)	0 (0.0%)	2 (1.1%)	
**Weekly physical activity**				
Not active	26 (29.9%)	15 (15.8%)	41 (22.5%)	0.009
Lightly active	36 (41.4%)	39 (41.1%)	75 (41.2%)	
Moderately active	16 (18.4%)	36 (37.9%)	52 (28.6%)	
Very Active	9 (10.3%)	5 (5.3%)	14 (7.7%)	
**Sleep hours per day**				
< 7	37 (42.5%)	32 (33.7%)	69 (37.9%)	0.356
7–9	45 (51.7%)	59 (62.1%)	104 (57.1%)	
> 9	5 (5.7%)	4 (4.2%)	9 (4.9%)	
**Social status**				
Single	86 (98.9%)	92 (96.8%)	178 (97.8%)	0.622
Engaged/married	1 (1.1%)	3 (3.2%)	4 (2.2%)	
**Smoking status**^a^				
Never	73 (83.9%)	73 (76.8%)	146 (80.2%)	0.547
Previous	2 (2.3%)	3 (3.2%)	5 (2.7%)	
Current	12 (13.8%)	19 (20.0%)	31 (17.0%)	

^a^Including Vape, hookah, etc.

**Fig. 1:**
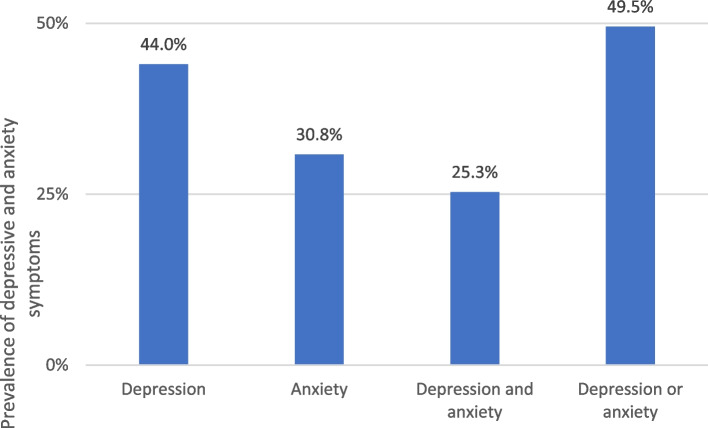
Prevalence of depressive and anxiety symptoms in all students (*n* = 182)

**Fig. 2:**
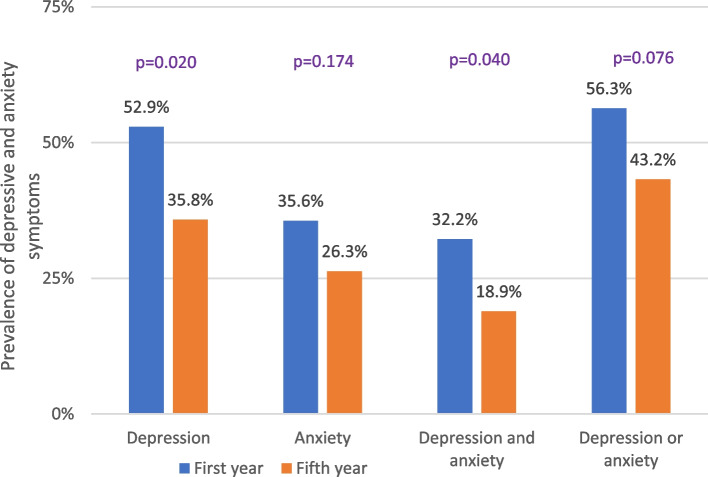
Prevalence of depressive and anxiety symptoms by the academic year of medical students (first-year *n* = 87, fifth-year *n* = 95)

As shown in Fig. 1, the prevalence of depressive and anxiety symptoms in all study students was 44.0% and 30.8%, respectively. Figure 2 shows that depressive symptoms were significantly higher in the first-year students than in the fifth-year students (52.9% versus 35.8%, *p* = 0.020). Anxiety symptoms were also higher in the first-year students than in the fifth-year students (35.6% versus 26.3%), but the difference did not reach statistical significance (*p* = 0.176). Approximately 25.3% of the students had both depressive and anxiety symptoms, and this prevalence was significantly higher in the first-year students than in the fifth-year students (32.2% versus 18.9%, *p* = 0.040). Approximately 49.5% of the students had either depressive or anxiety symptoms, without a significant difference between students in the two academic years (56.3% versus 43.2%, *p* = 0.076).

Table 2 shows the responses to COVID-19 pandemic-related questions by the academic year of students. Approximately one-fifth of the study students were always (9.9%) or most of the time (9.3%) worried about acquiring COVID-19, without significant differences between students in the two academic years (*p* = 0.517). Almost half of the study students were always (15.9%) or most of the time (33.5%) feeling worried or anxious about their academic performance during the COVID-19 pandemic, with significantly higher levels of worry in the first-year students than in the fifth-year students (63.2% versus 36.8%, *p* < 0.001). Approximately 30% of the study students always (8.8%) or most of the time (22.0%) felt sad, depressed or anxious during the COVID-19 pandemic, without significant differences between students in the two academic years (*p* = 0.648). Approximately 11.2% of the study students were diagnosed with COVID-19 (defined by a positive swab test), with a significantly lower infection rate in the first year than in the fifth year (2.4% versus 19.1%, *p* < 0.001). Half of the study students had at least one of their relatives diagnosed with COVID-19, without significant differences between students in the two academic years (*p* = 0.764).

**Table 2:** COVID-19 related questions by the academic year of medical students

	**First-year** **(*N* = 87)**	**Fifth-year** **(*N* = 95)**	**Total** **(*N* = 182)**	***p*-value**
**How often do you feel worried about acquiring COVID-19?**				
Not at all	26 (29.9%)	23 (24.2%)	49 (26.9%)	0.517
Sometimes	44 (50.6%)	54 (56.8%)	98 (53.8%)	
Most of the time	10 (11.5%)	7 (7.4%)	17 (9.3%)	
Always	7 (8.0%)	11 (11.6%)	18 (9.9%)	
**How often do you feel worried or anxious about your academic performance during COVID-19 pandemic?**				
Not at all	12 (13.8%)	17 (17.9%)	29 (15.9%)	< 0.001
Sometimes	20 (23.0%)	43 (45.3%)	63 (34.6%)	
Most of the time	32 (36.8%)	29 (30.5%)	61 (33.5%)	
Always	23 (26.4%)	6 (6.3%)	29 (15.9%)	
**How often do you feel sad, depressed or anxious during COVID-19 pandemic?**				
Not at all	18 (20.7%)	26 (27.4%)	44 (24.2%)	0.648
Sometimes	39 (44.8%)	43 (45.3%)	82 (45.1%)	
Most of the time	21 (24.1%)	19 (20.0%)	40 (22.0%)	
Always	9 (10.3%)	7 (7.4%)	16 (8.8%)	
**Have you been previously diagnosed with COVID-19?**				
No	82 (97.6%)	76 (80.9%)	158 (88.8%)	< 0.001
Yes	2 (2.4%)	18 (19.1%)	20 (11.2%)	
**Have any of your relatives been previously diagnosed with COVID-19?**				
No	41 (48.8%)	48 (51.1%)	89 (50.0%)	0.764
Yes	43 (51.2%)	46 (48.9%)	89 (50.0%)	

Table 3 shows the univariate and multivariate logistic regression analyses of potential predictors of depressive and anxiety symptoms. In addition to academic years, only the variables that were significantly (or almost significantly) associated with depressive or anxiety symptoms in univariate analysis are shown. First-year students were twice as likely to report depressive symptoms than fifth-year students in univariate analysis (odds ratio [OR] 2.01, 95% confidence interval [CI] 1.11–3.65, *p* = 0.021) but not in multivariate analysis. Independent risk factors for depressive symptoms included having concomitant anxiety (OR = 9.57, 95% CI 3.59–25.49, *p* < 0.001), being worried about acquiring COVID-19 (OR = 5.60, 95% CI 1.74–17.97, *p* = 0.004), being worried about academic performance (OR = 4.33, 95% CI 1.80–10.39, *p* = 0.001), and feeling sad, depressed or anxious (OR = 7.75, 95% CI 2.88–20.85, *p* < 0.001). Academic year was not a significant predictor of anxiety in either univariate or multivariate analysis. Independent risk factors for anxiety included having lower GPA (OR = 2.70, 95% CI 1.03–7.08, *p* = 0.044) and having concomitant depressive symptoms (OR = 13.91, 95% CI 6.08–31.81, *p* < 0.001).

**Table 3:** Univariate and multivariate^a^ logistic regression analysis of potential predictors of depressive and anxiety symptoms

	**Univariate analysis**				**Multivariate analysis**			
	Odds ratio (OR)	95% confidence interval of OR		*P*-value	Odds ratio (OR)	95% confidence interval of OR		*P*-value
		Lower	Upper			Lower	Upper	
**Depression**								
Academic year (first versus fifth)	2.01	1.11	3.65	0.021				
Psychological therapy (yes versus no)	2.34	1.13	4.86	0.023				
Sleep hours per day								
< 7 versus > 9	0.61	0.14	2.65	0.512				
7–9 versus > 9	0.27	0.06	1.12	0.071				
Anxiety (yes versus no)	12.45	5.66	27.40	< 0.001	9.57	3.59	25.49	< 0.001
COVID-19 related issues^b^								
Worried about acquiring infection	7.31	2.99	17.88	< 0.001	5.60	1.74	17.97	0.004
Worried about academic performance	5.77	3.03	10.99	< 0.001	4.33	1.80	10.39	0.001
Feeling sad, depressed or anxious	14.72	6.51	33.29	< 0.001	7.75	2.88	20.85	< 0.001
**Anxiety**	2.013	1.111	3.647	0.021				
Academic year (first versus fifth)	1.55	0.82	2.92	0.175				
GPA								
3.00 to 3.99 versus 4.50 to 5.00	2.24	0.97	5.17	0.059	2.28	0.84	6.18	0.105
4.00 to 4.49 versus 4.50 to 5.00	1.71	0.78	3.74	0.179	2.70	1.03	7.08	0.044
Psychological therapy (yes versus no)	2.18	1.04	4.56	0.038				
Sleep hours per day								
< 7 versus > 9	1.37	0.32	5.92	0.677				
7–9 versus > 9	0.63	0.15	2.72	0.538				
Depression (yes versus no)	12.45	5.66	27.40	< 0.001	13.91	6.08	31.81	< 0.001
COVID-19 related issues^b^								
Worried about acquiring infection	3.53	1.65	7.57	0.001				
Worried about academic performance	2.40	1.25	4.60	0.008				
Feeling sad, depressed or anxious	5.67	2.84	11.31	< 0.001				

^a^Multivariate logistic regression was done using backward elimination of all variables included in univariate analysis

^b^Always/most of the time versus not at all/sometimes

## Discussion

The current study revealed alarmingly high prevalence rates of depressive and anxiety symptoms among medical students during the COVID-19 pandemic. The current prevalence of depressive symptoms (44%) was generally similar to the prevalence reported by previous studies performed in Saudi Arabia and internationally [4, 6]. For example, a recent review that included 18 studies from different regions of Saudi Arabia estimated the prevalence of depression among medical students to be between 31 and 78%, with an average prevalence of 51% [4]. Interestingly, only three studies included in this review used the PHQ-9 in screening for depressive symptoms (similar to the current study), with slightly lower rates of depression that ranged between 28 and 61% [21–23]. It should be mentioned that all the included studies in that review were conducted before the COVID-19 pandemic. Consistent with the current finding, depressive symptoms detected in medical students in Western countries using different psychometric tools were estimated to be between 6 and 66% [6].

The current prevalence of anxiety symptoms (31%) was generally similar to the prevalence reported by previous studies performed in Saudi Arabia [24–26] and internationally [6, 24, 26, 27]. The anxiety rates in medical students in Saudi Arabia ranged between 28% and 66.3% [24–26]. This variability is largely caused by different study designs, use of different psychometric tools, and reporting different disease severities [24–26]. Consistent with the current finding, anxiety symptoms detected in medical students using different psychometric tools were estimated to be between 8 and 65% in Western countries before the COVID-19 pandemic and between 17 and 46% in developing countries after the COVID-19 pandemic [6, 27].

Both depressive and anxiety symptoms in the current study were higher in the first-year students than in the fifth-year students; this difference was significant for depressive symptoms. Similarly, some studies have shown that depressive symptoms are higher in new medical students than in clinical students, probably because of a lack of coping mechanisms with respect to the challenging new study environment [10, 11, 28]. Additionally, it has been shown that the prevalence of stress among medical students is highest among first-year students, diminishes progressively until the fourth year, and then slightly increases during the fifth year [29]. The only factor that was significantly higher in first-year students and was a significant predictor of depression was worry about academic performance during the COVID-19 pandemic. The current findings support the need for special mental health support programs targeting new medical students. This is especially important with closures, quarantine, and limited psychiatric services during the pandemic [30].

The current study showed that worrying about academic performance among medical students (49%) was much higher than worrying about contracting COVID-19 (19%). Additionally, COVID-19-related worries were independent risk factors for depressive and anxiety symptoms. This finding probably indicates the significant impact of the current COVID-19 pandemic on the mental health of medical students. Consistent with this finding, several international studies have reported the significant role of the current COVID-19 pandemic in developing depressive and anxiety symptoms among medical students [12–14]. Similar findings have been reported in Saudi Arabia [17]. Interestingly, an increase in depressive and anxiety symptoms during the COVID-19 pandemic has been observed in both medical and nonmedical students [31].

Data on types of worry during the COVID-19 pandemic and their relationship with mental health are very limited [13, 31]. There is differentiation between academic apprehension, which refers to worries about academic progress in the time of closures, and general apprehension, which refers to worries about the consequences of the disease on oneself, family and friends [13]. However, general but not academic apprehension was associated with depressive and anxiety symptoms in previous studies [13]. In response to the pandemic challenge, medical students can adopt different coping strategies to cope with the negative impact of the COVID-19 pandemic, including religious/spiritual coping and acceptance coping [32]. Additionally, strengthened family bonds during the lockdown were shown to improve mental health [33].

Our study is unique in quantifying the burden on the mental health of medical students in different academic years. We used validated psychometric tools to assess depressive and anxiety symptoms [18, 19]. Additionally, we examined the impact of COVID-19-related worries on depressive and anxiety symptoms. Nevertheless, a few limitations are acknowledged. Being a single institution study, the results should be generalized with caution. The cross-sectional design used cannot prove causation but rather association. Finally, the self-reported nature of the study data cannot exclude the possibility of recall bias. However, these limitations are present in almost all similar studies and are believed to have a very minor impact on the study findings, if any. We think this study highlights these worrisome findings; however, more qualitative studies need to be performed to further understand the risk factors for mental health disorders among medical students and identify potential measures to support this population.

## Conclusions

In conclusion, the current study shows an alarmingly high prevalence of depressive and anxiety symptoms among medical students during the COVID-19 pandemic. Both depressive and anxiety symptoms were significantly higher in the first-year students than in the fifth-year students, which reached statistical significance only for depressive symptoms. COVID-19-related worries were independent risk factors for depressive and anxiety symptoms. There is a need for special mental health programs targeting new medical students, especially those with limited psychiatric services during the pandemic.

## Supplementary Information

**Additional file 1. **

## Data Availability

The datasets used and/or analysed during the current study are available from the corresponding author on reasonable request.

## Abbreviations

PHQ-9: 9-Item Patient Health Questionnaire
GAD-7: 7-Item Generalized Anxiety Disorder assessment
DSM-IV: The Diagnostic and Statistical Manual of Mental Disorders
SD: Standard deviation
GPA: Grade point average

## References

[1] Vos T, Allen C, Arora M, Barber RM, Bhutta ZA, Brown A, et al. Global, regional, and national incidence, prevalence, and years lived with disability for 310 diseases and injuries, 1990–2015: a systematic analysis for the Global Burden of Disease Study 2015. The lancet. 2016;388(10053):1545–602. 10.1016/S0140-6736(16)31678-6 PMC5055577 27733282

[2] World Health Organization. Depression and other common mental disorders: global health estimates. World Health Organization; 2017.

[3] Baumeister H, Hutter N, Bengel J, Härter M (2011). Quality of life in medically ill persons with comorbid mental disorders: a systematic review and meta-analysis. Psychother Psychosom.

[4] AlJaber MI (2020). The prevalence and associated factors of depression among medical students of Saudi Arabia: A systematic review. J Family Med Prim Care.

[5] Elzubeir MA, Elzubeir KE, Magzoub ME (2010). Stress and coping strategies among Arab medical students: towards a research agenda. Educ Health (Abingdon).

[6] Hope V, Henderson M (2014). Medical student depression, anxiety and distress outside North America: a systematic review. Med Educ.

[7] Dyrbye LN, Thomas MR, Shanafelt TD (2006). Systematic review of depression, anxiety, and other indicators of psychological distress among U.S. and Canadian medical students. Acad Med.

[8] Dyrbye LN, Thomas MR, Harper W, Massie FS, Power DV, Eacker A, Szydlo DW, Novotny PJ, Sloan JA, Shanafelt TD (2009). The learning environment and medical student burnout: a multicentre study. Med Educ.

[9] Sawa RJ, Phelan A, Myrick F, Barlow C, Hurlock D, Rogers G (2006). The anatomy and physiology of conflict in medical education: a doorway to diagnosing the health of medical education systems. Med Teach.

[10] Alakhtar A, Al-Homaidan H (2014). Depression among medical students at qassim university rate, severity, and contributing factors; using BDI II. Int J Dev Res.

[11] Jarwan BK (2015). Depression among medical students of Faculty of Medicine, Umm Al-Qura University in Makkah, Saudi Arabia. Int J Med Sci Public Health.

[12] Bashir TF, Hassan S, Maqsood A, Khan ZA, Issrani R, Ahmed N, Bashir EF (2020). The Psychological Impact Analysis of Novel COVID-19 Pandemic in Health Sciences Students: A Global Survey. Eur J Dent.

[13] Saraswathi I, Saikarthik J, Senthil Kumar K, Madhan Srinivasan K, Ardhanaari M, Gunapriya R (2020). Impact of COVID-19 outbreak on the mental health status of undergraduate medical students in a COVID-19 treating medical college: a prospective longitudinal study. PeerJ.

[14] Ghazawy ER, Ewis AA, Mahfouz EM, Khalil DM, Arafa A, Mohammed Z, Mohammed EF, Hassan EE, Abdel Hamid S, Ewis SA, Mohammed AE (2020). Psychological impacts of COVID-19 pandemic on the university students in Egypt. Health Promot Int.

[15] Alkhamees AA, Alrashed SA, Alzunaydi AA, Almohimeed AS, Aljohani MS (2020). The psychological impact of COVID-19 pandemic on the general population of Saudi Arabia. Compr Psychiatry.

[16] Alamri HS, Algarni A, Shehata SF, Al Bshabshe A, Alshehri NN, ALAsiri AM, Hussain AH, Alalmay AY, Alshehri EA, Alqarni Y, Saleh NF (2020). Prevalence of Depression, Anxiety, and Stress among the General Population in Saudi Arabia during Covid-19 Pandemic. Int J Environ Res Public Health.

[17] Qanash S, Al-Husayni F, Alemam S, Alqublan L, Alwafi E, Mufti HN, Qanash H, Shabrawishi M, Ghabashi A (2020). Psychological Effects on Health Science Students After Implementation of COVID-19 Quarantine and Distance Learning in Saudi Arabia. Cureus.

[18] Kroenke K, Spitzer RL, Williams JB (2001). The PHQ-9: validity of a brief depression severity measure. J Gen Intern Med.

[19] Spitzer RL, Kroenke K, Williams JB, Lowe B (2006). A brief measure for assessing generalized anxiety disorder: the GAD-7. Arch Intern Med.

[20] American Psychiatric Association (1994). Diagnostic and statistical manual of mental disorders.

[21] Alsalameh NS, Alkhalifah AK, Alkhaldi NK, Alkulaib AA (2017). Depression among Medical Students in Saudi Arabia. Egypt J Hosp Med.

[22] Nooli A, Asiri A, Asiri A, Alqarni M, Alhilali F, Alayafi M (2017). Prevalence of depression among medical interns in King Khalid University. Int J Med Res Prof.

[23] Alshehri AA, Alaskar FA, Albahili FK (2018). Stress, Depression and Anxiety among Medical Students of Imam Mohammed Ibn Saud Islamic University, KSA. Egypt J Hosp Med.

[24] Al-Khani AM, Sarhandi MI, Zaghloul MS, Ewid M, Saquib N (2019). A cross-sectional survey on sleep quality, mental health, and academic performance among medical students in Saudi Arabia. BMC Res Notes.

[25] Aboalshamat K, Hou XY, Strodl E (2015). Psychological well-being status among medical and dental students in Makkah, Saudi Arabia: a cross-sectional study. Med Teach.

[26] Lateef Junaid MA, Auf A, Shaikh K, Khan N, Abdelrahim SA (2020). Correlation between Academic Performance and Anxiety in Medical Students of Majmaah University - KSA. J Pak Med Assoc.

[27] Lasheras I, Gracia-Garcia P, Lipnicki DM, Bueno-Notivol J, Lopez-Anton R, de la Camara C, Lobo A, Santabarbara J (2020). Prevalence of Anxiety in Medical Students during the COVID-19 Pandemic: A Rapid Systematic Review with Meta-Analysis. Int J Environ Res Public Health.

[28] Farrer LM, Gulliver A, Bennett K, Fassnacht DB, Griffiths KM (2016). Demographic and psychosocial predictors of major depression and generalised anxiety disorder in Australian university students. BMC Psychiatry.

[29] Abdulghani HM (2008). Stress and depression among medical students: A cross sectional study at a medical college in Saudi Arabia. Pak J Med Sci.

[30] Bojdani E, Rajagopalan A, Chen A, Gearin P, Olcott W, Shankar V, Cloutier A, Solomon H, Naqvi NZ, Batty N, Festin FE (2020). COVID-19 Pandemic: Impact on psychiatric care in the United States. Psychiatry Res.

[31] Saddik B, Hussein A, Sharif-Askari FS, Kheder W, Temsah MH, Koutaich RA, Haddad ES, Al-Roub NM, Marhoon FA, Hamid Q, Halwani R (2020). Increased Levels of Anxiety Among Medical and Non-Medical University Students During the COVID-19 Pandemic in the United Arab Emirates. Risk Manag Healthc Policy.

[32] Salman M, Asif N, Mustafa ZU, Khan TM, Shehzadi N, Tahir H, et al. Psychological impairment and coping strategies during the COVID-19 pandemic among students in Pakistan: a cross-sectional analysis. Disaster Medicine and Public Health Preparedness. 2022;16(3):920–6. 10.1017/dmp.2020.397 PMC7873451 33087206

[33] Alfawaz HA, Wani K, Aljumah AA, Aldisi D, Ansari MGA, Yakout SM, Sabico S, Al-Daghri NM (2021). Psychological well-being during COVID-19 lockdown: Insights from a Saudi State University's Academic Community. J King Saud Univ Sci.

