# Supplementary material for: The prevalence of depressive and anxiety symptoms among first-year and fifth-year medical students during the COVID-19 pandemic: a cross-sectional study
Source: BMC Med Educ. 2023 Jun 6;23:411. doi: 10.1186/s12909-023-04387-x (PMC10241552; doi:10.1186/s12909-023-04387-x)
Supplement: Supplementary file 1 — Additional file 1. [file 12909_2023_4387_MOESM1_ESM.pdf]

The Prevalence of Depressive and Anxiety Symptoms among First-Year and Fifth-Year Medical Students During the COVID-19 Pandemic: A Cross-Sectional Study

Dear Participants,

I would like to ask this opportunity if you are willing to take part of this questionnaire-based survey. Please answer the questions to the best of your knowledge. All information asked in this study questionnaire will be treated confidential. If you are willing to participate voluntarily in this study, please click “Yes, I do”

Do you agree to participate in this survey?

- Yes, I do
- No, I don’t

Sociodemographic

The First section of this survey will be directed towards personal information about you, the survey taker.

1- In which academic medical year are you?
- First year (1st)
- Fifth Year (5th)
- Other

2- Gender?
- Male ● Female

3- Age?
- (   )

4- What is your current GPA
- 4.50 to 5.00
- 4.00 to 4.49
- 3.50 to 3.99
- 3.00 to 3.49
- Less than 2.99

5- Number of failed academic years?
- None
- 1 Year
- 2 Years
- 3 Years

6- Are you currently undergoing any form of professional psychological therapy?
- Yes
- No

7- Who are you currently living with?
- Alone
- With family
- With roommate/s
- With spouse

8- How would you describe your weekly physical activity?
- Not active
- Lightly active
- Moderately active
- Very active

9- How many hours do you sleep daily? (on average)
- More than 9 hours
- 7-9
- 4-6
- Less than 4 hours

10- Are you currently…
- Married
- Single
- Divorced
- Engaged
- Widowed

11- Are you a smoker? (Including Vape, hookah, etc..)
- Yes
- No
- Previous smoker

12- How often do you feel worried about acquiring COVID-19?
- Not at all
- Sometimes
- Most of the time
- Always

13- How often do you feel sad, depressed or anxious during COVID-19 pandemic?
- Not at all
- Sometimes
- Most of the time
- Always

14- How often do you feel worried or anxious about your academic performance during COVID-19 pandemic?
- Not at all
- Sometimes
- Most of the time
- Always

15- Have you and/or any of your relatives been previously diagnosed with COVID-19?
- No
- I have been diagnosed with COVID-19
- Relative diagnosed with COVID-19
- Both

PHQ-9

The second section of this study is a validated PHQ-9 scale of depressive symptoms, which consists of 9 questions in total.

1- Little interest or pleasure in doing things
- Not at all
- Several days
- More than half of the days
- Nearly everyday

2- Feeling down, depressed, or hopeless
- Not at all
- Several days
- More than half of the days
- Nearly everyday

3- Trouble falling or staying asleep, or sleeping too much
- Not at all
- Several days
- More than half of the days
- Nearly everyday

4- Feeling tired or having little energy
- Not at all
- Several days
- More than half of the days
- Nearly everyday

5- Poor appetite or over-eating
- Not at all
- Several days
- More than half of the days
- Nearly everyday

6- Feeling bad about yourself or that you are a failure or have let yourself or your family down
- Not at all
- Several days
- More than half of the days
- Nearly everyday

7- Trouble concentrating on things, such as reading the newspaper or watching television
- Not at all
- Several days
- More than half of the days
- Nearly everyday

8- Moving or speaking so slowly that other people could have noticed. Or the opposite being so fidgety or restless that you have been moving around a lot more than usual
- Not at all
- Several days
- More than half of the days
- Nearly everyday

9- Thoughts that you would be better off dead or of hurting yourself
- Not at all

- Several days
- More than half of the days
- Nearly everyday

GAD-7

The third and final section of this study is validated GAD-7 scale that measures anxiety, this section is composed of 7 questions in total.

1-Feeling nervous, anxious or on an edge

- Not at all
- Several days
- More than half of the days
- Nearly everyday

2-Not being able to stop or control worrying

- Not at all
- Several days
- More than half of the days
- Nearly everyday

3- Worrying too much about different things

- Not at all
- Several days
- More than half of the days
- Nearly everyday

4- Trouble relaxing

- Not at all
- Several days
- More than half of the days
- Nearly everyday

5- Being so restless that it's hard to sit still

- Not at all
- Several days
- More than half of the days
- Nearly everyday

6- Becoming easily annoyed or irritable

- Not at all
- Several days
- More than half of the days
- Nearly everyday

7- Feeling afraid, as if something awful might happen *

- Not at all
- Several days
- More than half of the days
- Nearly everyday

Thank you for your participation, your response has been submitted.

Have a wonderful day.
